# The Homeopathic Materia Medica

**Published:** 1897-04

**Authors:** Rufus Choate

**Affiliations:** Washington, D. C.


### THE HOMEOPATHIC MATERIA MEDICA.

Rufus Choate, M. D., Washington, D. C.

Homeopathy has the distinction of possessing a materia medica based upon certain recognized laws and principles that characterize a science. The materials used in the homeopathic practice of medicine adapt themselves to their laws. In this respect Homeopathy is a science. Established upon these principles Homeopathy has a materia medica that distinguishes it from all other schools of medicine. In as far as the principles are observed Homeopathy is practiced, and when these principles are abandoned the science of Homeopathy suffers. As you know, the fundamental law of our science is that a disease can be cured by the drug-material or cause, that has in its effects an image of the effects of the disease. To learn these effects of the drug provings were made by persons recognized not to be affected by a disease similar to the action of the drug. Occasionally in proving a drug a symptom of disease, of which the prover was not aware was similar to the drug's action, disappeared and a clinical proving resulted. These clinical provings are of great importance and have given aid in the selection of the remedy by which life frequently has been saved. A great discovery was made which revolutionized the administration of remedies. It is that the power of the drug in the cure of disease is wonderfully increased by potentiation. The homeopathic physician need not potentiate his drug. He is at liberty to give it in its crude form, or he may go to the highest possible attenuation and keep his title clear, but he cannot give an unproven remedy or a combination of remedies and be an homeopath. The successful physician can use other means than those belonging to our materia medica. He can, if he so desires, administer morphine for pain; he can attempt the alleviation of disease by electricity; he can call to his aid the skilled surgeon, but just so far as he deviates from the law that makes Homeopathy a science, just so far he ceases to be an homeopath.

Is there necessity for an increase of our materia medica? For the next twenty years we have an abundance of material. If we are to keep our science pure we must protest against the introduction of fads, of newly-advertised unproven remedies and cure-alls. The remedies proven are already so great in number, the symptoms recorded are so vast the physician will find material enough to carry him through nearly every emergency without the addition of many new drugs. The tendency to grasp the new is the acknowledgment that we have not thoroughly studied the old. The most important thing to learn of a drug is its genius. Every drug, as every man, or every thing, has its peculiar genius that gives it individuality. It is not the multiplicity of symptoms that should be committed to memory any more than it is the everchanging gestures of a man that should be studied. It is the heart and brain behind the symptoms, or gestures, which once learned will foretell them. He is a remarkable man who obtains a knowledge of the genius of four drugs in a year; he has the capacity that may make him a master in Homeopathy who has this knowledge of many drugs. Without this knowledge you are a novice, though a hundred years old. Ask yourself how many drugs you know so intimately that you can recognize their appropriate application in symptoms not yet recorded. Obtain this knowledge and you can take Allen's able, though unsystematized, *Encyclopedia*, and erase hundreds of symptoms which have no bearing upon the drug under which they are placed. I advise every beginner in the science to make a deep, thorough, unceasing study of the old, proven, long-used remedies of our materia medica. Give no thought to new remedies. Let the old hands try these for you, prove them, and if they approve them, consider them carefully and jealously watch against encroachments. Finally, demand their proving prior to using. To protect our materia medica I advocate the establishment in each medical society of a committee of three, to be known as the Society Committee on Remedies. The duty of these committees should be to take up and consider every remedy that enters our materia medica. They should have no authority to certify any remedy that has not had a certain amount of provings, nor should they be permitted to consider any remedy other than that assigned by the Medical Society of which the committee is a part. Each committee should select a chairman, who, joined with the other chairmen of the other committees, should form a Special Committee on Remedies. They should receive the remedies acted upon by each Society Committee, and after passing upon them should certify them to the general profession. In this way our materia medica would obtain an authority and a purity that is now much endangered.

It is hardly necessary to enumerate the many valuable remedies that make our materia medica rich, but I deem it proper to examine one that has lately obtained much attention at your hands. To our theraphy electricity has been added with an intelligence and corroborated by results so confirmatory of benefit that it becomes only right to do homage to those skilled in its application. When it was broached there were doubts in the minds of some with reference to its rapidly advancing application, which tended or appeared to tend to the subordination of the appropriate homeopathic remedy, as to whether it was in accordance with the science of Homeopathy. It became evident that as far as electricity is used so far Homeopathy is not used, and the conclusion follows that, thus far, the physician curing cases, as he asserts he does, by electricity, ceases to be an homeopath. The homeopath may blunder and fail in the selection of his remedy, but his very failures tend, if he holds fast to the law, to raise him to a full appreciation of his science. He who fails and rises again fails that he may rise to higher things; he who succeeds, no higher ascends.

There is a force, as yet but little studied, through which cures are made, life is given, the tree grows and the human body exists. This force is magnetism. When a substance, speech, or thought of one plane meets a perfect receptacle on another plane a mighty force is produced, and these two substances become, as it were, one. The human mind finds its perfect bed in the human body, and the two are so intimately conjoined that seeing one we see the other. The subjective medicine meets its exact similar, the objective disease-state, and a life-giving, curative magnetic force results. As long as the conjunction continues there is an efflux of magnetism, and separation of the conjunction is death. Electricity is the separator. There was a perfect marriage which electricity sundered. The magnetic hand is the warm, pulsating palm of life; the electric is the skeleton hand pointing to death. I earnestly advise the physician to look carefully at the path he selects and to see whether it is well to produce decomposition, separation, and death, which have the appearance of life and give the appearance of cure, but are, in fact, dissolution.

In conclusion, I warn the beginner in Homeopathy that he attempts the acquisition of a science. He will not master the knowledge of the greatest curative art given to humanity within a four years' study. In fact, to become a master in Homeopathy I am of the opinion, without the egotism to presume to know, that it takes twenty-five years of close, unremitting application. Do not begin with the higher potencies. The use of high potencies will come with the knowledge of the genius of the drug, and not safely without this knowledge. He who begins at the top of the ladder advances downward. Do not be disappointed if the remedies you apply under the law of Homeopathy do not perform a cure. Let us illustrate: There is a certain most excruciating headache. It is not difficult to find a remedy having a similar headache in its provings, but no drug will cure that headache. Why? Simply because it is caused by a tight shoe pressing a sensitive corn. Remove the pressure and the headache will disappear. In other words, use common sense and cultivate observation. I had the honor at a previous meeting of this society to assert that symptoms should not be immediate guides to the remedy. I reiterate the statement. He who treats on symptoms only travels an endless chain, and will find confusion his reward. Let the symptom guide to the cause, and this removed the symptoms disappear. Again poverty, or family disgrace, or danger of impending failure of business will produce certain disease-states. Give little hope of your skill in prescribing until the cause is removed. Finally, we come to the appreciation that the mind is a great factor in the case. As with medicines, so with diseases, *ad interna accretio potentis est.*

